# Carboxyhemoglobin, a reliable diagnosis biomarker for hemolysis in intensive care unit: a retrospective study

**DOI:** 10.1186/s13054-020-03437-w

**Published:** 2021-01-05

**Authors:** Geoffroy Hariri, Kyann Hodjat Panah, Bénédicte Beneteau-Burnat, Michael Chaquin, Arsene Mekinian, Hafid Ait-Oufella

**Affiliations:** 1 Assistance Publique – Hôpitaux de Paris (AP-HP), Service de réanimation médicale, Hôpital Saint-Antoine, 184 Rue du Faubourg Saint-Antoine, 75571 Paris Cedex 12, France; 2 Sorbonne Université, Université Pierre-et-Marie Curie, Paris 6, France; 3 Assistance Publique – Hôpitaux de Paris (AP-HP), Service de biochimie, Hôpital Saint-Antoine, 75571 Paris Cedex 12, France; 4 Assistance Publique – Hôpitaux de Paris (AP-HP), Laboratoire d’hématologie, Hôpital Saint-Antoine, 75571 Paris Cedex 12, France; 5 Assistance Publique – Hôpitaux de Paris (AP-HP), Service de médecine interne, Hôpital Saint-Antoine, 75571 Paris Cedex 12, France; 6 Inserm U970, Centre de Recherche Cardiovasculaire de Paris (PARCC), Paris, France

## Dear Editor,

Hemolytic anemia (HA) is a common condition in intensive care units (ICU) responsible for life-threatening organ failure in severe cases [1]. HA needs urgent treatment initiation, but its diagnosis remains challenging as none of the biological diagnostic parameters, including bilirubin, LDH and haptoglobin, are specific. During hemolysis, free hemoglobin released by red blood cells is catabolized by heme-oxygenase 1, leading to formation of iron, biliverdin and carbon monoxide [2]. Next, carbon monoxide binds to free hemoglobin to form carboxyhemoglobin. Carboxyhemoglobin is routinely measured in ICU and available within a few minutes by CO-oximetry, a point of care testing (GEM® Premier™ 4000, Werfen, Le Pré-Saint-Gervais, France) [3]. Our objective was to evaluate carboxyhemoglobin as a diagnostic tool for HA in adult patients admitted in ICU.

## Methods and statistical analysis

We retrospectively analyzed data from patients hospitalized for HA and non-HA in our ICU during an 8-year and 1-year period, respectively. An adjudication committee consisting of three senior experts (from hematology, internal medicine and critical care departments) confirmed final HA and non-HA diagnosis with all available clinical and biological data. Carboxyhemoglobin was measured at ICU admission on arterial blood.

Differences between groups were compared using the Student's *t* test or Wilcoxon’s test. Correlations were computed using Pearson’s formula. Discrimination performances were assessed by using area under the receiver operating characteristic curve (ROC). Statistical analyses were performed using R software (v 2.12.0; http://cran.r-project.org).

## Results

Overall, 187 patients were included, 94 patients with HA and 93 with non-HA (Table 1). Among patients with HA, 50 (54%) had thrombotic micro-angiopathy, 25 (26%) had auto-immune hemolytic anemia, and 19 (20%) had sickle cell disease.

**Table 1:** Characteristics of patients with and without hemolytic anemia

	Hemolytic anemia (94)	Non-hemolytic anemia (93)	*p*
General characteristics			
Age (years)	47 (32–62)	69 (59–77)	0.0004
Women (%)	51	33	0.004
Smokers (%)	34	37	0.9
SOFA at admission	6 (3–7)	6 (4–9)	0.004
Sepsis (%)	18	27	0.1
Mechanical ventilation (%)	17	57	< 0.0001
Catecholamines (norepinephrine) (%)	17	54	< 0.0001
Extra-renal epuration (%)	17	13	0.43
Length of stay in ICU (days)	6 (3–12)	7 (4–12)	0.54
Mortality (%)	16	32	0.24
Cause of anemia (%)			
Hemolytic anemia (94)			
AIHA	26		
SCD	20		
TMA	54		
Non-hemolytic anemia (93)			
Bleeding		37	
Chronic renal failure		15	
Cytopenia		23	
Splenomegaly		10	
Other		15	
Biological marker (blood)			
Hemoglobin (g/dL)	7.8 (6.2–9.1)	7.3 (6.5–8.1)	0.14
White blood cells (× 10^9^/L)	10.6 (7.8–17.9)	11.3 (6.7–16.5)	0.2
Platelets (10 ^3^/mm^3^)	76 (33.2–228)	141 (48–237)	0.09
Mean corpuscular volume (fL)	88 (83–93)	88 (82–94)	0.47
Reticulocytes (G/L)	134 (77–211)	55 (29–77)	0.0001
LDH (UI/L)	1361 (866–2121)	536 (420–786)	0.46
Unconjugated bilirubin (μmol/L)	27 (18–45)	15 (10–20)	< 0.0001
Prothrombin time (%)	76 (62–89)	70 (57–80)	0.009
Activated partial thromboplastin time ratio	1.07 (0.94–1.3)	1.17 (1.01–1.51)	0.055
Creatinine (μmol/L)	102 (71–160)	122 (81–206)	0.47
pH	7.44 (7.4–7.48)	7.44 (7.34–7.48)	0.25
pO2 (mmHg)	87 (70–113)	81 (67–107)	0.92
pCO2 (mmHg)	34 (31–43)	35 (30–42)	0.93
Lactate (mmol/L)	1.3 (0.9–2.5)	1.25 (0.9–2.3)	0.88
Carboxyhemoglobin (%)	3.0 (2.3–3.9)	1.6 (1.0–2.1)	< 0.0001
Methemoglobin (%)	1.5 (1.2–1.8)	1 (0.7–1.4)	< 0.0001

Data are expressed as number, percentage or median and interquartile ranges (IQRs)

*SOFA* sepsis-related organ failure assessment, *AIHA* auto-immune hemolytic anemia, *TMA* thrombotic micro-angiopathy, *SCD* sickle cell disease, *LDH* lactate dehydrogenase

**Fig. 1:**
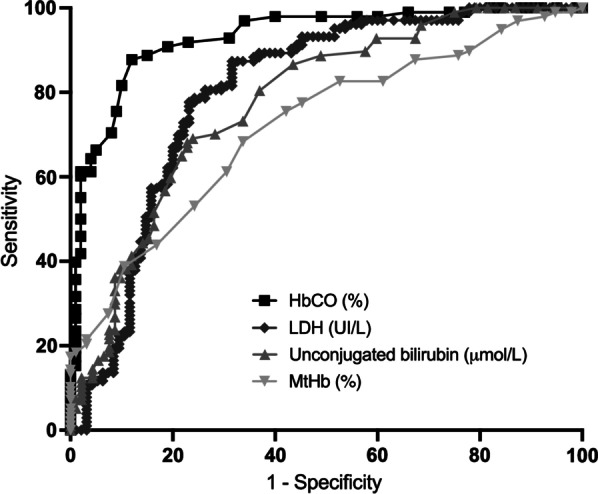
Receiver operator characteristic curves for hemolysis biomarkers: carboxyhemoglobin (HbCO) (black square), AUC = 0.93 (CI 95% [0.89–0.96]); lactate dehydrogenase (gray diamond), AUC = 0.80 (CI 95% [0.73–0.86]), unconjugated bilirubin (light gray triangle), AUC = 0.77, (CI 95% [0.71–0.84]) and methemoglobin (reversed light gray triangle), AUC = 0.71 (CI 95% [0.64–0.79])

Carboxyhemoglobin levels were twofold higher in patients with HA in comparison with patients with non-HA (3.0 [2.3–3.9] vs 1.6 [1.0–2.1] %, *p* < 0.0001). Carboxyhemoglobin level at admission was an accurate diagnostic tool for HA as the area under the curve (AUC) was 0.93 (CI 95% [0.89–0.96]), higher than LDH (AUC = 0.80, CI 95% [0.73–0.86]), unconjugated bilirubin (AUC = 0.77, CI 95% [0.71–0.84]) and methemoglobin (AUC = 0.71, CI 95% [0.64–0.79]) (Fig. 1). A threshold of carboxyhemoglobin of 2.0% for detection of hemolysis, yielded a sensitivity of 85% (CI 95% [77–90]) and specificity of 86% (CI 95% [80–90]). Specificity of carboxyhemoglobin for hemolysis detection was ≥ 99% for levels ≥ 2.7%. Using a logistic regression, we adjusted the analysis of carboxyhemoglobin for age and SOFA and we found that carboxyhemoglobin remained strongly associated with hemolysis: crude OR 74 (CI 95% [19–281]), adjusted OR 53 (CI 95% [12–240], *p* < 0.001 (both Wald and LR test). Focusing on patients with HA, we found that carboxyhemoglobin levels inversely correlated with hemoglobin levels (*r* = 0.42, *p* < 0.0001).

## Discussion

In anemic adult patients admitted in ICU, we found that carboxyhemoglobin was a reliable diagnostic biomarker of hemolysis. Diagnostic accuracy of HA was better using carboxyhemoglobin than LDH and unconjugated bilirubin, when an optimal threshold of 2.0% was used. A similar threshold of carboxyhemoglobin at 2.2% has recently been reported in a cohort of term newborns [4]. In critically ill patients with comorbidities and multiple organ failures, classical hemolysis biomarkers as LDH and unconjugated bilirubin may lack specificity [5]. Haptoglobin is another biomarker for hemolysis, but its level may change in several critical conditions including sepsis or red blood cell transfusion [6]. Unfortunately, in our study, haptoglobin was not available in patients with non-HA. We also found a significant relationship between plasma carboxyhemoglobin and hemoglobin levels, meaning that the higher the carboxyhemoglobin, the more severe the hemolytic anemia. Carboxyhemoglobin has to be analyzed after evaluation of confounding factors that potentially increase (heavy smoker, sepsis, carbon monoxide chronic sub-intoxication) or decrease (hyperoxia) its levels.

## Data Availability

The datasets used and/or analyzed during the current study are available from the corresponding author on reasonable request.
